# Detection of 65 kD heat shock protein in cerebrospinal fluid of tuberculous meningitis patients

**DOI:** 10.1186/1471-2377-6-34

**Published:** 2006-09-15

**Authors:** Anju V Mudaliar, Rajpal S Kashyap, Hemant J Purohit, Girdhar M Taori, Hatim F Daginawala

**Affiliations:** 1 Biochemistry Research Laboratory, Central India Institute of Medical Sciences, Nagpur – 440 010, India; 2 Environmental Genomics Unit, National Environmental Engineering Research Institute, Nehru Marg, Nagpur-440020, India; 3 Central India Institute of Medical Sciences, 88/2 Bajaj Nagar, Nagpur-440010, India.

## Abstract

**Background:**

Diagnosis of tuberculous meningitis (TBM) is difficult. Rapid confirmatory diagnosis is essential to initiate required therapy. There are very few published reports about the diagnostic significance of 65 kD heat shock protein (hsp) in TBM patients, which is present in a wide range of *Mycobacterium tuberculosis *species and elicits a cellular and humoral immune response. In the present study we have conducted a prospective evaluation for the demonstration of 65 kD hsp antigen in cerebrospinal fluid (CSF) of TBM patients, by indirect ELISA method using monoclonal antibodies (mAb) against the 65 kD hsp antigen, for the diagnosis of TBM.

**Methods:**

A total of 160 CSF samples of different groups of patients (confirmed TBM {n = 18}, clinically suspected TBM {n = 62}, non TBM infectious meningitis {n = 35} and non-infectious neurological diseases {n = 45}) were analyzed by indirect ELISA method using mAb to 65 kD hsp antigen. The Kruskal Wallis test (Non-Parametric ANOVA) with the Dunnett post test was used for statistical analysis.

**Results:**

The indirect ELISA method yielded 84% sensitivity and 90% specificity for the diagnosis of TBM using mAb to 65 kD hsp antigen. The mean absorbance value of 65 kD hsp antigen in TBM patients was [0.70 ± 0.23 (0.23–1.29)], significantly higher than the non-TBM infectious meningitis group [0.32 ± 0.14 (0.12–0.78), *P *< 0.001] and also higher than the non-infectious neurological disorders group [0.32 ± 0.13 (0.20–0.78), *P *< 0.001]. A significant difference in the mean absorbance of 65 kD hsp antigen was noted in the CSF of culture-positive TBM patients [0.94 ± 0.18 (0.54–1.29)] when compared with clinically suspected TBM patients [0.64 ± 0.20 (0.23–0.98), *P *< 0.05].

**Conclusion:**

The presence of 65 kD hsp antigen in the CSF of confirmed and suspected cases of TBM would indicate that the selected protein is specific to *M. tuberculosis *and could be considered as a diagnostic marker for TBM.

## Background

The world health organization estimates that there are more than 8 million new cases of tuberculosis (TB) each year [1,2]. Among tuberculosis, tuberculous meningitis (TBM) has become more common with the emergence of human immunodeficiency virus (HIV) infection [3,4]. TBM leads to multiple central nervous system (CNS) complications and if untreated remains a major health problem in underdeveloped and developing countries. Diagnosis of TBM remains problematic despite many new advanced diagnostic methods. A reliable and rapid diagnostic test, which can be performed in any standard pathology laboratory, can be of help in early definitive diagnosis of TBM.

A number of studies have reported the use of various methodologies for demonstration of *Mycobacterium tuberculosis *antigens and antibodies in cerebrospinal fluid (CSF) of TBM patients. Most of the antigens used in these studies were either cell wall components or culture filtrate proteins of *M tuberculosis *[5-8]. However, there are very few published reports to detect the heat shock protein (hsp) in CSF for diagnosis of TBM.

Various families of Hsps like Hsp90, Hsp70, Hsp65 and Hsp10 have been shown to elicit strong immune responses in the host during tuberculous infection [9,10]. Amongst these, one particular antigen 65 kD hsp (Rv0440) is present in wide range of *Mycobacterium tuberculosis *species and is immunodominant which elicits cellular and humoral immune responses [11-13]. This protein is produced in response to host reaction during infection and thus the more general term, stress protein has been applied to this class of proteins [14]. 65 kD hsp plays a dual role in cells, primarily as molecular chaperones and also as immunodominant antigens upon infection in the host [10]. A study indicated that the level of these proteins of *M. tuberculosis *increases by 1–10% under conditions of stress which is likely to occur during tuberculous infection [15].

The quantitative measurement of 65 kD hsp antigen levels in CSF samples of TBM patients may provide new insights into the diagnostic role of 65 kD hsp in TBM infection. In the present study, we have conducted a prospective laboratory investigation for the detection of 65 kD hsp antigen, in CSF of TBM patients by indirect ELISA method using monoclonal antibodies (mAb) against the 65 kD hsp antigen.

## Methods

A total of 160 CSF samples of different group of patients, which includes TBM (confirmed cases = 18, clinically suspected cases = 62), non-TBM infectious meningitis (pyogenic meningitis = 25, viral meningitis = 10) and non-infectious neurological disorders (n = 45, which includes cases of stroke, headache etc) were analyzed in the present study. Patients included in the study are those admitted to the Neurology Department of Central India Institute of Medical sciences (CIIMS). 65 kD hsp antigen estimation was done in the CSF samples obtained before starting any treatment in all cases of neurological disorders including viral, bacterial, and mycobacterial meningitis. All patients were grouped as follows:

### Tuberculosis Meningitis (n = 80)

#### Confirmed cases (n = 18)

Presence of *Mycobacterium tuberculosis *in CSF by culture

#### Clinically suspected (n = 62)

This group included culture negative cases with all of the following observations.

a) Sub-acute or chronic fever with features of meningeal irritation such as headache, neck stiffness and vomiting with or without other features of CNS involvement b) CSF findings showing increased proteins, decreased glucose (CSF: blood glucose ratio <0.5), and/or pleocytosis with lymphocytic predominance c) Good clinical response to antituberculous drugs. None of these TBM patients had positive AFB staining.

#### Non-TBM infectious meningitis (n = 35)

This group included patients of pyogenic and viral meningitis.

### Pyogenic meningitis (n = 25)

#### Confirmed cases (n = 4)

Presence of pathogenic bacteria such as *Staphylococcus sps*, *Streptococcus sps, Haemophilus sps *in CSF by staining and/or culture

#### Clinically suspected (n = 21)

This group included culture negative cases with all the following observations:

a) Fever and/or signs of meningeal irritation (patients who have undergone cranial surgery to treat tumor(s), stroke, or head injury and who have received antibiotics), OR High fever and/or signs of meningeal irritation with or without other CNS manifestations (patients who received broad-spectrum antibiotics). b) CSF findings showing increased proteins, decreased glucose (CSF: blood glucose ratio <0.2), and/or pleocytosis with a predominance of polymorphonuclear cells; c) Good clinical response to broad-spectrum antibiotics

#### Viral meningitis (n = 10)

Mainly caused by Enterovirus and Herpesvirus. This group included suspected patients with following observations:

a) Acute onset of fever and symptoms and signs of meningeal irritation. b) CSF findings showing mild increase in protein, glucose often normal and pleocytosis, predominantly lymphocytic c) No clinical evidences of extra cranial tuberculosis.

#### Non-infectious neurological disorders (n = 45)

-All other patients who had no evidence of CNS or extra CNS bacterial or viral infections were grouped in the non-infectious/control group. Patients included in this group are of chronic intractable headache, status epileptics, stroke etc.

The study was approved by the Institutional Ethics Committee of CIIMS, Nagpur.

### Specimen

CSF sample was collected by standard lumbar puncture. Approximately 3 ml of CSF was obtained. It was used for total and differential cell count, biochemistry, and smear for Gram's, India ink, and AFB staining and for detection of 65 kD hsp antigen by indirect ELISA. All the samples were stored at -20°C until further analysis.

### Antibodies

The 65 kD monoclonal antibody to 65 kD hsp antigen was obtained from Colorado State University, USA under the TB Research Materials and Vaccine Testing Contract (NO1-AI-75320) derived from *Mycobacterium tuberculosis*, strain H_37_Rv, designated IT13. The secondary antibody was rabbit anti rat obtained from Genei, Bangalore, India.

### One-dimensional PAGE

CSF samples obtained from TBM and non TBM cases were subjected to Sodium dodecyl sulfate polyacrylamide gel electrophoresis (SDS-PAGE). SDS-PAGE was performed with a vertical slab gel electrophoresis system (Broviga, India) using the standard Laemmali method, [16]. 4% stacking gel and 10% running gel were used. Electrophoresis was carried out at 250 volts/50 mAmps. Gels were developed by staining with Coomassie brilliant blue GR-250 and the protein profiles were then studied. Band size (i.e., molecular weight) was estimated using molecular weight markers (Genei, Bangalore, India).

### Immunoblotting assay

CSF proteins (35ug/lane) were separated by SDS-PAGE, and transferred to Polyvinyledineflouride (PVDF) memberane by electroblotting at 100 V for 3 hours The membrane was wetted in 50%v/v methanol prior to electroblotting and after electroblotting. The membrane was then blocked with 0.5% bovine serum albumin (BSA) in phosphate buffer saline (PBS) and 0.05% Tween 20 (PBS-T) for 60 min at 37°C. After blocking, the membrane was washed with PBS-T (3times 10min each), and probed with monoclonal antibody (1:2000 dilution in PBS-T) generated against 65 kD hsp antigen and incubated at 37°C for 60 min. The membrane was then washed with PBS-T, followed by addition of affinity purified anti-rat IgG conjugated to horseradish peroxidase(Genei, Bangalore, India) diluted 1:10,000 in PBS-T, and incubated at 37°C for 60 min After incubation the membrane was washed extensively with PBS-T followed by addition of tetramethylbenzidine-hydrogen peroxide (TMB/H_2_O_2_)as substrate and the antibody reactivity was visualized.

### Indirect ELISA for detection of 65 kD hsp antigen

Prior to patients sampling, the assay was standardized using different concentration of 65 kD antigen (1–1000ng/ml) in PBS-T (pH 7.2). After standardization, wells of flat-bottom microtiter plates were coated with 100 ùl of CSF samples (1:5 dilution in PBS-T) of selected groups and incubated for 90 min at 37°C. The wells were then washed with PBS-T and blocked with 100 ùl of 0.5% BSA in PBS-T at 37°C for 60 min. After blocking, monoclonal antibody generated against 65 kD hsp antigen was added to all the wells (1:5,000 dilution in PBS-T) and incubated at 37°C for 60 min. The wells were washed with the PBS-T followed by addition of, 100 ùl of affinity purified anti-rat IgG conjugated to horseradish peroxidase(Genei, Bangalore, India) with 1:10,000 dilution in PBS-T, and incubated at 37°C for 60 min. After incubation the wells were washed extensively with PBS-T followed by addition of 100ul of TMB/H_2_O_2 _substrate and incubated at room temperature for 10 min. The reaction was stopped with addition of 100 ùl of 2.5 N H_2_SO4. The absorbance of each well was read at 450 nm. Each sample was tested in triplicate.

### Statistical analysis

Results are expressed as mean ± SD with range. To compare mean absorbance value of 65 kD hsp antigen among the TBM, non-TBM infectious meningitis and non-infectious neurological disorders, the Kruskal Wallis test (Non-Parametric ANOVA) with the Dunnett post test was used. A *P *value less than 0.05 was considered significant. A cut off value of absorbance of 65 kD hsp antigen for TBM patients was calculated using the mean plus SD of the absorbance of 65 kD hsp antigen in the non-TBM neurological disorders group. The sensitivity (true positive rate) for the test was calculated as: [the number of samples in the TBM group with absorbance ≥ (mean + SD) of absorbance in the non-infectious neurological disorders group divided by the total number of samples in the TBM group] × 100. The specificity (true negative rate) for the test was calculated as: [the number of samples in the non-TBM group with absorbance < (mean + SD) of the absorbance in the non-infectious neurological disorders group divided by the total number of samples in the non-TBM group] × 100.

## Results

The CSF samples of 160 patients admitted to the Neurology Department of CIIMS hospital were grouped into three different categories (TBM, non-TBM infectious meningitis and non infectious neurological disorders) on the basis of clinical observations and biochemical and pathological analyses of CSF samples. Of these 18 were of confirmed TBM, 62 of suspected TBM, 35 of non-TBM infectious meningitis {pyogenic meningitis (25) and viral meningitis (10)} and 45 of non-infectious neurological disorders.

**Figure 1:**
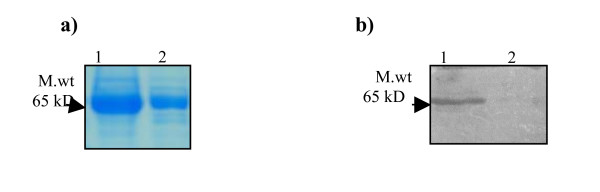
One-dimensional PAGE and Immunoblot. Fig 1a SDS-PAG electrophoretogram of CSF samples. Lane (1) CSF of TBM patient, lane (2) CSF of non-TBM patient. Fig 1b Immunoblot analysis using specific rabbit antibodies against 65 kD antigen. Lane (1) CSF of TBM patient, lane (2) CSF of non-TBM patient.

The CSF proteins were separated (35ug total protein in each lane) by SDS-PAGE and blotted on to PVDF membrane. Figure. 1 depicts the one-dimensional PAGE and Immunoblot analysis. Fig. 1a SDS-PAG electrophoretogram of CSF samples. CSF of TBM patient (lane 1), shows more protein expression as compared to CSF of non TBM patient (lane 2). Fig 1b depicts immunoblotting with specific rabbit antibodies against 65 kD hsp antigen. The CSF of TBM patient (lane 1) shows reactivity for 65 kD hsp antigen, which was absent in case of non-TBM patient(lane 2).

**Figure 2:**
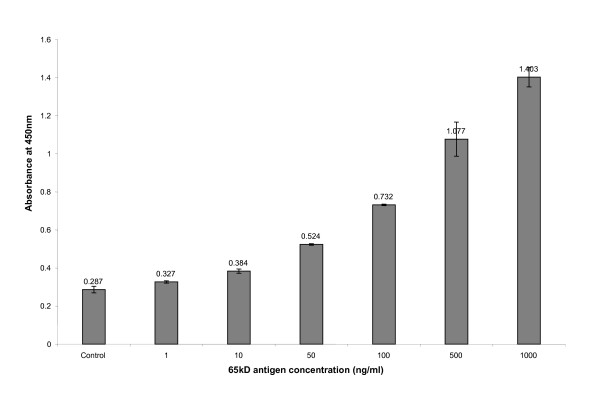
Absorbance at 450 nm with increasing concentration of 65 kD antigen in the standardization procedure for indirect ELISA method.

Different concentrations of 65 kD hsp antigen were titrated with its monoclonal antibody and a standard graph was plotted with the increasing concentration of antigen. Figure. 2 show the increase in absorbance at 450 nm with increasing concentration of 65 kD hsp antigen during the standardization of indirect ELISA method.

Table 1 depicts the demonstration of 65 kD hsp in CSF of patients of TBM, non-TBM infectious meningitis and non infectious neurological disorders by indirect ELISA method using mAb specific to 65 kD hsp antigen. The CSF positivity for 65 kD hsp antigen in case of confirmed and clinically suspected patients was 100% (18/18) and 77% (48/62) respectively, while the positivity for patients with pyogenic meningitis was 8% (2/25) and viral meningitis was 10% (1/10). In the non infectious neurological disorders group, 11% (5/45) of patients had CSF positivity for 65 kD hsp antigen. Overall the indirect ELISA method yielded 84% sensitivity and 90% specificity for the diagnosis of TBM using mAb to 65 kD hsp antigen. Cut-off value was calculated using mean ± Standard deviation of control group.

Table 2 depicts the mean absorbance with range and interquartile range of the 65 kD hsp antigen in the CSF of TBM, non TBM infectious and non TBM non infectious patients. The data are expressed as mean ± Standard Deviation. The mean absorbance value of 65 kD hsp antigen in TBM patients was [0.70 ± 0.23 (0.23–1.29)] significantly higher than the non-TBM infectious meningitis group [0.32 ± 0.14 (0.12–0.78); *P *< 0.001]and also higher than the non-infectious neurological disorders group [0.32 ± 0.13 (0.20–0.78); *P *< 0.001].

**Figure 3:**
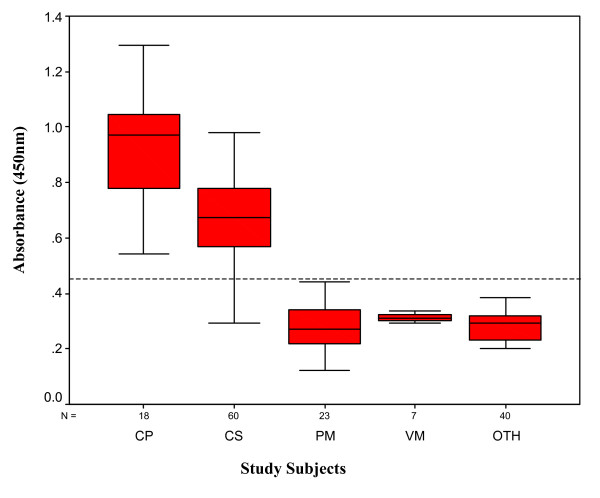
Box plot for demonstration of 65 kD hsp in CSF of culture positive (CP) and clinically suspected (CS) TBM patients, non-TBM infectious meningitis (PM- pyogenic meningitis; VM-viral meningitis) and non infectious neurological disorders groups (OTH). The box plot show 5^th ^and 95th percentiles (bars), 75th and 25^th ^percentiles (boxes) and median (bars in boxes). N – numbers of individual in each group.

Figure 3 shows the box plot for demonstration of 65 kD hsp antigen in CSF of culture positive and clinically suspected TBM patients, non-TBM infectious meningitis patients and non infectious neurological disorders groups. The box plot shows 5^th ^and 95th percentiles (bars), 75th and 25^th ^percentiles (boxes) and median (bars in boxes). N -numbers of individual in each group.

## Discussion

TBM is often difficult to diagnose because of its diverse clinical presentations which can mimic other clinical neurological disorders like pyogenic meningitis, particularly partially treated, other CNS infectious and non infectious disorders [17,18]. Early confirmatory diagnosis of TBM is difficult to establish because of its pleomorphic clinical presentation [19,20]. Delayed diagnosis and treatment may be associated with many serious CNS complications [21]. The most commonly used laboratory method for the definitive diagnosis of TBM is to demonstrate the presence of tubercle bacilli either by smear and/or culture. However, direct smear methods are often negative in CSF samples and culturing of MTB takes 4–6 weeks [22,23]. Recent methods such as those involving the amplification of bacterial DNA by the polymerase chain reaction and comparable systems are incompletely assessed and not available for widespread use in the developing countries. The sensitivity of the PCR technique varies from 33% to 90% and the specificity from 88% to 100% [24]. Various immunoassays such as antigen and/or antibody detection in CSF samples have been developed with variable sensitivities and specificities [25-30]. Despite extensive work on TBM, only few diagnostic tests are available [23,25,28]. A reliable and rapid diagnostic test, which can be performed in any standard pathology laboratory, can be of help in diagnosis of TBM.

The 65 kD hsp antigen has received a great deal of attention recently because it appears to be one of the major immunologically active mycobacterial antigens following infection and is expressed at high levels by bacterial pathogens during adaptation for intracellular survival. In spite of the interest generated in *M.tuberculosis *specific 65 kD hsp, only limited attempts have been made to study this protein in the diagnosis of tuberculous meningitis.

In the present study we have evaluated the 65 kD hsp antigen activity in CSF samples obtained from patients of TBM, non TBM infectious meningitis and non-infectious neurological diseases, using monoclonal antibody against 65 kD hsp antigen by indirect ELISA method. The data of this study demonstrates 100% and 77% positivity in the CSF of culture-positive and clinically-suspected TBM patients, respectively. False positive results were noted in 8 % of pyogenic meningitis and 10% of viral meningitis cases. Overall the indirect ELISA method yielded 84% sensitivity and 90% specificity for the diagnosis of TBM using mAb to 65 kD hsp antigen.

Various families of hsp have been shown to elicit strong immune responses in the host. Among these, the Hsp70 and Hsp65 classes have shown to play key roles in eliciting immune responses [10]. Besides the diagnostic importance, this protein seems to have important role in pathogenesis of tuberculosis. In a recent study Azov AG et al[31] developed nested polymerase chain reaction (PCR) protocols for detecting a *Mycobacterium *genus-specific 65 kD hsp sequence and the *M. tuberculosis *complex-specific insertion sequence IS6110 in formalin-fixed and paraffin-embedded sections with good sensitivity and specificity[32]. To our knowledge there is no report of diagnostic importance of this protein detection by ELISA in CSF specimen. 65 kD hsp antigen detection using indirect ELISA method is sensitive, specific, rapid and cost effective, and could find practical application even in laboratories with limited resources and technical expertise.

## Conclusion

Our data suggests that the detection of 65 kD hsp antigen in CSF of TBM patients can be useful for early diagnosis of TBM. Detection of 65 kD hsp antigen using indirect ELISA method is cost effective and could be an alternative to other more expensive sophisticated techniques.

## Competing interests

The author(s) declare that they have no competing interests.

## Authors' contributions

AVM carried out the study design, data collection, statistical analysis, data interpretation, literature search, and manuscript preparation; RSK and HJP participated in the preparation of the manuscript, data interpretation, and study design; GMT provided assistance in preparation of the manuscript, data interpretation, study design, and funds collection; and HFD supervised the study design, statistical analysis, data interpretation, manuscript preparation, and literature search. All authors have read and approved the final version of the manuscript.

## Pre-publication history

The pre-publication history for this paper can be accessed here:



## Figures and Tables

**Table 1:** Demonstration of 65 kD antigen in CSF of TBM, non TBM infectious and non TBM non infectious patients.

**Groups**	**Positivity for 65 kD hsp protein**	**Negativity for 65 kD hsp protein**
**TBM (n = 80)**	**66 (84%)**	**14 (16%)**
Suspected (n = 62)	48 (77%)	14 (23%)
Confirmed (n = 18)	18 (100%)	-
		
**Non-TBM (80)**	**8 (10%)**	**72 (90%)**
		
**Non TBM infectious meningitis (n = 35)**	**3 (14 %)**	**32 (86%)**
Viral meningitis (n = 10)	01(10%)	09 (90%)
Pyogenic meningitis(n = 25)	02 (8%)	23 (92%)
		
**Non-infectious neurological Disorders (45)**	**5 (11%)**	**40 (89%)**
GBS(n = 12)	01	11
Stroke(n = 10)	04	06
Migraine(n = 08)	-	08
Encephalopathy(n = 10)	-	10
Fever(n = 05)	-	05

**Table 2:** Demonstration of mean absorbance with range and interquartile range of 65 kD antigen in the CSF of TBM, non TBM infectious and non TBM non infectious patients. The data are expressed as mean ± SD.

**Patient group**	**Absorbance Mean ± SD**	**Range**	**Interquartile Range**
1. Tuberculous Meningitis (n = 80)	0.70 ± 0.23	0.23 – 1.29	0.57–1.05
			
Culture Positive (n = 18)	0.94 ± 0.18	0.54 – 1.29	0.78–1.05
Clinically Suspected (n = 62)	0.64 ± 0.20	0.23 – 0.98	0.57–0.78
			
2. Non TBM infectious meningitis (n = 35)	0.32 ± 0.14	0.12 – 0.78	0.22–0.35
			
Pyogenic meningitis (n = 25)	0.32 ± 0.16	0.12 – 0.78	0.22–0.35
Viral meningitis (n = 10)	0.33 ± 0.09	0.20 – 0.56	0.30–0.34
			
3. Non-infectious neurological disorders (n = 45)	0.32 ± 0.13	0.20 – 0.78	0.23–0.34
